# Alterations in functional connectivity analyzed using MREG in patients with suspected autoimmune psychosis spectrum syndromes

**DOI:** 10.1016/j.bbih.2025.101111

**Published:** 2025-09-22

**Authors:** Katharina von Zedtwitz, Ludger Tebartz van Elst, Isabelle Matteit, Andrea Schlump, Thomas Lange, Kimon Runge, Judith Weiser, Kathrin Nickel, Katharina Domschke, Harald Prüss, Alexander Rau, Marco Reisert, Simon J. Maier, Bernd Feige, Dominique Endres

**Affiliations:** a Department of Psychiatry and Psychotherapy, Medical Center - University of Freiburg, Faculty of Medicine, University of Freiburg, Freiburg, Germany; b Division of Medical Physics, Department of Diagnostic and Interventional Radiology, Medical Center - University of Freiburg, Faculty of Medicine, University of Freiburg, Freiburg, Germany; c German Center for Mental Health (DZPG), Partner Site Berlin/Potsdam, Berlin, Germany; d Department of Neurology and Experimental Neurology, Charité - Universitätsmedizin Berlin, Berlin, Germany; e German Center for Neurodegenerative Diseases (DZNE), Berlin, Germany; f Department of Neuroradiology, Medical Center - University of Freiburg, Faculty of Medicine, University of Freiburg, Freiburg, Germany; g Department of Stereotactic and Functional Neurosurgery, Faculty of Medicine, Medical Center - University of Freiburg, University of Freiburg, Freiburg, Germany

**Keywords:** Autoimmune encephalitis, Antibody, Neuroinflammation, fMRI, Resting state

## Abstract

**Introduction:**

In NMDA-R encephalitis, which is typically accompanied by psychotic symptoms, conventional magnetic resonance imaging (MRI) is often normal, despite widespread alterations in functional connectivity. This is the first functional connectivity study in psychiatric patients with suspected autoimmune psychosis (AP) spectrum syndromes.

**Methods:**

Twenty-eight patients with suspected AP spectrum syndromes who were selected according to the concept of autoimmune psychiatric syndromes (APS) and 28 matched healthy controls (HCs) were examined with ultrafast functional MRI using magnetic resonance encephalography (MREG). Patients were positive for either well-characterized or novel central nervous system antibodies or well-characterized systemic antibodies with autoimmune brain involvement. MREG data were processed using “Analysis of Functional NeuroImages” (AFNI) with the “Functional And Tractographic Connectivity Analysis AFNI toolbox” to analyze connectivity across 170 regions, yielding an analysis of 5995 evaluable connectivities.

**Results:**

After correction for multiple testing, functional connectivity between the left middle cingulate/paracingulate gyri and the right insula (p_adj_ = 0.025) was significantly reduced in the patient group compared to HCs. Exploratory analyses revealed widespread global functional connectivity alterations in 226 of all connections (corresponding to 3.8 %). Notably, of these altered connections, 99 % showed reduced connectivity, while 1 % showed hyperconnectivity. The medial pulvinar of the left thalamus emerged as the most disconnected hub with altered connectivity to 33 other regions. Overall, 46 % of all analyzed regions exhibited at least one altered functional connectivity, with 19 % of hubs located in the cerebellum, 11 % in the frontal brain, and 9 % in the thalami. After correction for multiple comparisons, increased connectivity between the left insula and the left superior temporal gyrus correlated with the Beck Depression Inventory scores (p_adj_ = 0.043).

**Discussion:**

Patients with suspected AP spectrum syndromes exhibit altered insular functional connectivity associated with the severity of depressive symptoms. Broader changes identified via hypothesis-generating analyses highlighted major hubs in the cerebellum, frontal brain, and thalamus. These findings suggest that functional MRI may serve as an additional tool for detecting patients with AP/APS. Future studies in more homogeneous autoimmune-mediated patient groups may help delineate specific connectivity signatures in functional networks.

## 1 Introduction

The initial description of anti-N-methyl-D-aspartate receptor (NMDA-R) encephalitis in 2007, which often manifests with additional psychotic symptoms (Dalmau et al., 2007; Dalmau et al., 2008; Dalmau et al., 2019), has drawn attention to autoimmune-mediated secondary psychosis. In recent years, several neuronal or glial antibodies have been identified as being associated with autoimmune encephalitis (AE) and additional psychiatric symptoms (Dalmau and Graus, 2018; Pollak et al., 2020; Prüss, 2021; Guasp and Dalmau, 2024; Li et al., 2024). International consensus criteria for the diagnosis of AE (Graus et al., 2016) and autoimmune psychosis (AP) have been published (Pollak et al., 2020). Additional autoimmune psychiatric syndromes (APS), sometimes encompassing mixed psychiatric syndromes (Herken and Prüss, 2017; Endres et al., 2022a, 2022b), have also been described, and the concept of APS has been proposed (Al-Diwani et al., 2017; Hansen et al., 2020; Tebartz van Elst et al., 2025). These developments have significantly influenced neuroimmunological research and clinical practice in neurology and, increasingly, in psychiatry over the last two decades. In psychiatric patients, a spectrum of novel central nervous system (CNS) antibodies has recently been identified in cerebrospinal fluid (CSF); however, the exact target antigens and their functionality remain to be studied (Endres et al., 2022c). Routine diagnostics for AE/AP/APS typically include conventional magnetic resonance imaging (MRI) of the brain, electroencephalography (EEG), CSF analysis, and testing for CNS antibodies in serum and CSF (Graus et al., 2016; Endres et al., 2020a; Pollak et al., 2020; Tebartz van Elst et al., 2025). In addition, [^18^F]fluorodeoxyglucose positron emission tomography (FDG-PET) of the brain can be used in uncertain diagnostic cases to detect encephalitis, while whole-body FDG-PET can help to rule out tumors in paraneoplastic forms (Probasco et al., 2017; Bacchi et al., 2018; Bordonne et al., 2021; Tebartz van Elst et al., 2025). Conventional MRI often reveals inconspicuous findings or only non-specific changes, even in severe diseases such as NMDA-R encephalitis (MRI abnormalities in 33 %) or LGI1 encephalitis (MRI abnormalities in 37 %) (Titulaer et al., 2013; van Sonderen et al., 2016a, 2016b).

Advanced neuroimaging techniques offer promising insights in such cases. Morphometric analyses can detect volumetric brain changes (e.g., von Zedtwitz et al., 2025a, von Zedtwitz et al., 2025b), while magnetic resonance spectroscopic imaging (MRSI) studies can identify changes in neurochemistry (e.g., Endres et al., 2025), and functional magnetic resonance imaging (fMRI) can assess functional resting-state connectivity. Initial studies using fMRI in AE cohorts have demonstrated large-scale abnormalities (Peer et al., 2017; Heine et al., 2018; Hartung et al., 2024). A PubMed search for corresponding fMRI studies in AP/APS using the search terms “(autoimmune psychosis OR autoimmune psychiatric syndrome) AND (functional MRI OR fMRI OR resting-state connectivity OR MREG OR MR-encephalography)” performed on March 29, 2025, yielded 239 results but no available resting-state functional connectivity case-control studies. The fMRI methodology is based on the principle that anatomically distinct brain regions are functionally connected and exhibit time-dependent neuronal activation patterns that facilitate information exchange (Handwerker and Bandettini, 2011; Sirotin et al., 2009). Therefore, fMRI measures neuronal activity in the brain by detecting the differential magnetic susceptibilities of oxyhemoglobin and deoxyhemoglobin using the low-frequency blood oxygen level-dependent (BOLD) signal (van den Heuvel and Hulshoff Pol, 2010). The technique of magnetic resonance encephalography (MREG) is an ultrafast fMRI approach for optimal mapping of changes in hemodynamic activity (Hennig et al., 2021). Although the BOLD response is relatively slow, MREG allows the separation of BOLD effects from pulsatility caused by the heartbeat or breathing; this results in a higher sensitivity for the identification of dynamic variability of resting state networks or localization of interictal events in patients with epilepsy or epilepsy-related processes (Hennig et al., 2021).

***The rationale of this study*** was to investigate functional connectivity in patients with suspected AP spectrum syndromes compared to healthy controls (HCs). Initial studies using fMRI in AE cohorts — in which the patients often have accompanying psychotic or neurocognitive symptoms — have demonstrated large-scale functional connectivity abnormalities (Peer et al., 2017; Heine et al., 2018; Hartung et al., 2024). It was hypothesized that patients with suspected AP spectrum syndromes would exhibit disrupted functional networks, consistent with fMRI findings in NMDA-R encephalitis, where psychotic symptoms were particularly associated with changes in functional connectivity of frontoparietal networks (Peer et al., 2017). Methodologically, the ultrafast fMRI technique of MREG was used. As the first functional connectivity study in a suspected AP-spectrum syndrome cohort, this work reports results corrected for multiple testing while also presenting exploratory uncorrected findings to facilitate hypothesis generation.

## 2 Methods and participants

This project was part of a transdiagnostic and multimodal brain imaging study. Approval was obtained from the Ethics Committee of the University Medical Center Freiburg, Germany (Application No. EK-Freiburg: 209/18). Written informed consent was provided by all participants prior to enrollment. The study cohort has been partially described in previous research on brain morphometry (von Zedtwitz et al., 2025b) and MRSI findings (Endres et al., 2025).

### 2.1 Patient and control group assessment

***Patients*** were recruited from the Department of Psychiatry and Psychotherapy at the University Medical Center Freiburg, Germany. Eligible patients whose initial diagnosis occurred within the last 10 years were offered participation in this study with further advanced MRI and EEG measurements and a broad psychometric/neuropsychological test battery. Adult patients (age ≥18 years) were included if they tested positive for well-characterized neuronal antibodies (e.g., against the NMDA-R) (Endres et al., 2020b) or had novel CNS antibodies in a tissue-based assay on unfixed mouse brain slices (e.g., against vessels or granule cells) (for examples see von Zedtwitz et al., 2025b; Kreye et al., 2020). In addition, patients with well-characterized systemic antibodies (i.e., antinuclear antibodies [ANAs] measured on human embryonic kidney cells or thyroid antibodies) and clear signs of brain involvement in further diagnostic testing (i.e., who were diagnosed as neuropsychiatric lupus or Hashimoto encephalopathy; Graus et al., 2016; Legge and Hanly, 2024) were included. As part of the diagnostic routine workup, patients underwent CSF analysis, conventional MRI, EEG, and serum and CSF antibody testing. Well-characterized neuronal antibodies against intracellular antigens were tested in serum by immunoblot (Ravo®, Freiburg, Germany) and against cell surface antigens in serum and CSF using a fixed, cell-based assay (Euroimmun®, Lübeck, Germany). In addition, most patients were tested for neuronal antibodies using a tissue-based assay with unfixed mouse brain tissue (Kreye et al., 2020). Many patients also underwent cerebral FDG-PET (Endres et al., 2020b) as part of the clinical diagnostic work-up. The inclusion criteria for the suspected AP spectrum syndrome patient group were defined broadly following the “APS-approach” (Al-Diwani et al., 2017; Herken and Prüss, 2017; Hansen et al., 2020; Tebartz van Elst et al., 2025). Accordingly, not only patients with acute-onset first episode paranoid hallucinatory psychosis, but also patients with chronic courses and with predominant affective/neurocognitive/obsessive-compulsive syndromes were studied. Patients were classified as predominant schizophreniform psychoses or predominant affective spectrum syndromes. If neurocognitive symptoms were present in addition to psychosis or as predominant syndrome, patients were assigned to the “predominant schizophreniform psychoses subgroup”. Obsessive-compulsive syndromes in combination with affective syndromes were grouped under the “predominant affective spectrum syndrome subgroup” (as was one severe isolated predominant obsessive-compulsive syndrome with pre-diagnosed comorbid depressive syndrome earlier in the course). The focus was therefore on the suspected common autoimmune causality. 1) Acutely ill, 2) chronically ill and 3) (partially) remitted patients were studied. Prior immunotherapy or the use of psychopharmacological medication did not preclude participation.

***Healthy controls*** were ≥18 years of age and free of current or previous mental illness. Recent drug use within the last six months (except sporadic cannabis use) or any lifetime-use of psychopharmacological drugs led to exclusion from the study. Somatic diseases (e.g., inflammatory diseases such as systemic lupus erythematosus) or risk factors (e.g., head trauma) with possible brain involvement also resulted in exclusion. The screening approach for the control group was described in detail in two earlier studies (Endres et al., 2025; von Zedtwitz et al., 2025b).

***MRI-related exclusion criteria*** for the patients and HC group included pregnancy, lactation, lack of legal capacity or understanding of the study's nature, significance, and scope, and MRI contraindications (claustrophobia, intrauterine contraceptive devices, pacemakers, metallic foreign objects etc.).

### 2.2 Psychometric/neuropsychological test battery, additional EEG analyses, and matching

**Fig. 1:**
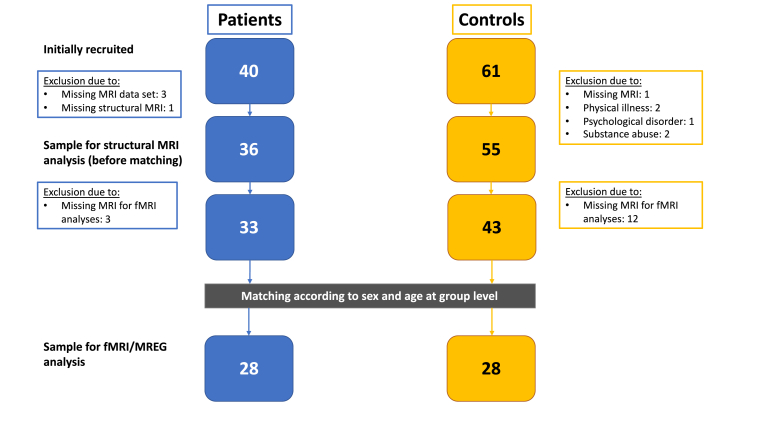
**Recruitment flowchart (the structural and MR-spectroscopy data of the patient group have already been published**; Endres et al., 2025; von Zedtwitz et al., 2025b**).** Abbreviations: fMRI, functional magnetic resonance imaging; MREG, magnetic resonance encephalography; MRI, magnetic resonance imaging.

A comprehensive set of sociodemographic, psychometric and neuropsychological tests was administered, as described in detail elsewhere (Endres et al., 2025; von Zedtwitz et al., 2025b). Incomplete data sets did not generally lead to exclusion from the study if the above-mentioned inclusion and exclusion criteria were met. This was particularly helpful for some patients who were unable to complete all the questionnaires or neuropsychological tests due to their illness. Clinical EEGs were available for all subjects and analyzed for intermittent rhythmic delta/theta activity (IRDA/IRTA) using independent component analysis (ICA) with an in-house software (https://github.com/berndf/avg_q). IRDA/IRTA rates with a lenient threshold of >1 μV were reported as events per minute before hyperventilation (HV) and as the difference between after and before HV (IRDA-difference) (Endres et al., 2017; von Zedtwitz et al., 2025a, von Zedtwitz et al., 2025b). Patients and controls were matched by age and sex. Automated matching was done using the “cardinality” method in the “MatchIt” R package with tolerances of 2 years of age and 0.2 for sex matching (Ho et al., 2011). In total, 28 matched pairs of patients and HCs were included in the final analysis. The complete recruitment process is summarized in the flowchart in Fig. 1.

### 2.3 MRI measurement and analyses

MRI scans were acquired using a Magnetom Prisma 3-T system (Siemens Healthineers®, Erlangen, Germany). A 64-channel head and neck coil was used for signal reception. During the scan, respiratory activity was measured with a breathing belt, and cardiac activity was monitored with a 4-channel electrocardiography (ECG) (both from Siemens facilities). MREG was used to achieve fMRI data with ultrafast acquisition with minimal loss of spatial resolution (Zahneisen et al., 2012; Assländer et al., 2013). All patients were asked to look at a white cross presented on a BOLDScreen MRI-compatible monitor via a small mirror during the MREG investigation. MREG allows 3D whole-brain imaging with an echo time of 33 ms, and a repetition time (TR) of 100 ms with a flip angle set to 21°. This ultrafast fMRI technique uses multi-channel parallel acquisitions with fast 3D k-space trajectories and iterative image reconstruction (Hennig et al., 2021). After reconstruction, volume registration was performed using custom MATLAB code, writing six motion parameters into a file for later regression and censoring. Further MRI processing was performed using “Analysis of Functional NeuroImages” (AFNI) software (Cox, 1996). After normalizing the anatomical image using “sswarper2”, the “afni_proc.py” meta-script was used for the fMRI preprocessing steps despiking, alignment, “tlrc” registration to normalized anatomy, masking, blurring with 4 mm full width at half maximum (FWHM), and scaling (normalizing to % signal change). In a regression step, motion parameters as obtained during volume registration and its derivative as well as 13 parameters for heartbeat and respiration phase and amplitudes (physio_calc.py; Birn et al., 2006) were regressed out. Functional connectivity was computed using the “Functional And Tractographic Connectivity Analysis AFNI toolbox” (FATCAT; Taylor and Saad, 2013) to perform Fisher z-transformed correlations between all pairs of the 170 regions from the automated anatomical labelling (AAL3v1) atlas (Rolls et al., 2020). In principle, 14365 connectivities would therefore be possible. However, automated AAL regions with missing data are sorted out, i.e. regions that are located beyond the mask that are not covered by the fMRI field of view (FOV) in all or some data sets. Finally, 5995 connectivities could be analyzed.

### 2.4 Statistical analyses

Statistical analyses were performed using the R software (v.3.6.0; R Foundation for Statistical Computing Platform, Vienna, Austria). Group comparisons for categorical variables (e.g., sex) were conducted using Fisher's exact test or Chi-square tests. A *t*-test for independent samples was used for dimensional variables (e.g., age). The Fisher z-transformed fMRI connectivities for each available region pair were analyzed with a linear model including group, sex and mean-centered age as independent variables. A lenient uncorrected significance threshold of p < 0.01 was applied for a first data reduction step. Hubs were identified by determining the number of altered connectivities, on this lenient significance level, involving each brain region. To find connections related to a range of clinical/psychometric measures across the suspected AP spectrum syndrome group, Fisher z-transformed fMRI connectivities for each available region pair were analyzed using a linear model including one psychometric score as dependent variable, and sex and mean-centered age as independent variables. Psychometric scores included the Positive and Negative Syndrome Scale (PANSS), the Eppendorf Schizophrenia Inventory (ESI), and the Beck Depression Inventory-II (BDI-II). Clinical information, such as automated EEG-slowing rates (IRDA/IRTA rates before HV and IRDA/IRTA differences) and dimensional CSF variables [white blood cell (WBC) count, protein levels, albumin quotient (AQ), and immunoglobulin G (IgG) index], were also analyzed. Again, hubs were identified using region pairs with a significance of the psychometric score influence of p < 0.01. Multiple testing correction was applied using the Benjamini–Hochberg approach (Benjamini and Hochberg, 1995), with a significance level of p < 0.05.

## 3 Results

### 3.1 Patient and control group

The study included 28 patients (mean age of 38.1 ± 13.1 years, 46 % female) and 28 healthy controls (HC). No significant differences in age (p = 0.896) or sex (p = 0.593) were observed between groups. Among the patients, 54 % had predominant affective spectrum syndromes, while 46 % had predominant schizophreniform psychotic syndromes. Well-characterized antibodies were detected in 21 % of patients, whereas 79 % tested positive for novel CSF antibodies. Antibodies were present in the CSF in 86 % of cases. Clinical MRI alterations were identified in 79 % of patients (excluding non-specific white matter changes and anatomical variants such as benign cystic lesions, alterations in 18 % were found), CSF pathologies in routine testing in 50 %, cerebral FDG-PET hyper/hypometabolism in 47 % (data available for 19 patients), and EEG abnormalities on visual inspection in 32 % (Table 1). Psychopharmacological medication was administered to 93 % of patients, with 61 % receiving atypical antipsychotics and 39 % receiving antidepressants. The HC group did not receive any psychopharmacological treatment. Regarding disease progression, 29 % of patients were in an acute stage, 39 % in a chronic stage, and 32 % were (partially) improved. Overall, 61 % of patients received immunotherapies (after multidisciplinary case discussions) before MRI scanning. A comprehensive summary of diagnostic findings and treatments is provided in Table 1. Psychometric and neuropsychological testing revealed significantly higher scores in the suspected AP spectrum syndrome group on the PANSS, ESI (for attention and speech impairment and deviant perception impairment), BDI-II, Autism Spectrum Quotient, attention-deficit/hyperactivity disorder checklist (ADHD-CL), Symptom Check List 90 (SCL-90), Spielberger's State-Trait Anxiety Inventory-Global (STAI-G), and Wender Utah Rating Scale (WURS). The patient group scored significantly lower on the Culture Fair Intelligence Test 20-Revised (CFT20-R). Several significant differences were also identified in the Test of Attentional Performances (TAP) and the Verbal Learning Memory Test (VLMT), with the suspected AP spectrum group generally scoring lower than HCs (Table 2).

**Table 1:** Characteristics and routine diagnostic findings of the suspected autoimmune psychosis spectrum syndrome group. Only the predominant antibody was mentioned if several antibodies were positive.

	**Patients (N=28)**		**Patients (N=28)**
**Antibody findings**			

**Well-characterized anti-CNS antibodies (N=28)**	6 (21 %)		
**In CSF**	2 (7 %)	**In Serum**	5 (18 %)
Anti-LGI1	0 (0 %)	Anti-LGI1	0 (0 %)
Anti-NMDA-R	1 (3.6 %)	Anti-NMDA-R	0 (0 %)
Anti-MOG	0 (0 %)	Anti-MOG	1 (3.6 %)
Anti-Yo	0 (0 %)	Anti-Yo	1 (3.6 %)
Anti-CASPR2	0 (0 %)	Anti-CASPR2	1 (3.6 %)
Anti-VGCC	0 (0 %)	Anti-VGCC	1 (3.6 %)
Anti-TPO	0 (0 %)	Anti-TPO	0 (0 %)
ANAs	1 (3.6 %)	ANAs	1 (3.6 %)
**Novel anti-CNS autoantibodies (N=28)**	22 (79 %)		
**In CSF**	21 (75 %)	**In Serum**	12 (43 %)
Anti-astrocytic pattern	1 (3.6 %)	Anti-astrocytic pattern	1 (3.6 %)
Anti-vessel pattern	5 (17.9 %)	Anti-vessel pattern	2 (7.1 %)
Anti-granule cell pattern	2 (7.1 %)	Anti-granule cell pattern	1 (3.6 %)
Anti-cytoplasmic pattern	1 (3.6 %)	Anti-cytoplasmic pattern	2 (7.1 %)
Anti-glia cell pattern	1 (3.6 %)	Anti-glia cell pattern	0 (0 %)
Anti-axon initial segment pattern	1 (3.6 %)	Anti-axon initial segment pattern	1 (3.6 %)
Anti-perinuclear pattern	3 (10.7 %)	Anti-perinuclear pattern	2 (7.1 %)
Anti-myelin pattern	3 (10.7 %)	Anti-myelin pattern	3 (10.7 %)
Anti-Purkinje cell pattern	2 (7.1 %)	Anti-Purkinje cell pattern	0 (0 %)
Anti-hippocampal pattern	2 (7.1 %)	Anti-hippocampal pattern	0 (0 %)

**Clinical information**			

**Syndrome overall**		**Clinical state**	
Predominant affective spectrum syndrome	15 (54 %)*	Acute state	8 (29 %)
Predominant schizophreniform psychotic syndrome	13 (46 %)**	(Partial) remission	9 (32 %)
* 4 patients with (additional) obsessive-compulsive syndromes, 3 patients with additional neurocognitive symptoms** 7 patients with additional affective syndromes, 1 patient with additional obsessive-compulsive syndrome, no patient with additional neurocognitive symptoms		Chronic disease (>2 years)	11 (39 %)
**Current psychotropic medication**		**Previous psychotropic medication**	
Antidepressants	11 (39 %)	Antidepressants	19 (68 %)
Typical Antipsychotics	4 (14 %)	Typical Antipsychotics	2 (7 %)
Atypical Antipsychotics	17 (61 %)	Atypical Antipsychotics	14 (50 %)
Anticonvulsants	3 (11 %)	Anticonvulsants	8 (29 %)
Mood Stabilizers	6 (21 %)	Mood Stabilizers	3 (11 %)
Benzodiazepines	3 (11 %)	Benzodiazepines	7 (25 %)
**Overall**	**26 (93 %)**	**Overall**	**21 (75 %)**

**Diagnostic findings**			

**CSF findings (N=28)**		**Clinical EEG pathologies (N=28)**	
Increased WBC counts	4 (14 %)	Focal slowing	1 (3.6 %)
Increased albumin quotients	8 (29 %)	Intermittent generalized slowing	8 (29 %)
Increased protein concentrations	10 (36 %)	Continuous generalized slowing	0 (0 %)
Increased IgG index	2 (7 %)	Epileptic activity	0 (0 %)
OCB in serum	0 (0 %)	**Overall**	**9 (32 %)**
OCB in CSF	2 (7 %)		
**Overall**	**14 (50 %)**		
**Clinical routine MRI (N=28)**		**FDG-PET pathologies (N=19)**	
Non-specific white matter changes	20 (71 %)	Hypermetabolism	5 (26 %)
(Chronic) inflammatory lesions	0 (0 %)	Hypometabolism	5 (26 %)
Global atrophy	4 (14 %)	**Overall**	**9 (47 %)**
Focal atrophy	4 (14 %)		
Pineal cyst	6 (21 %)		
Others	5 (18 %)		
**Overall**	**22 (79 %)**		

Abbreviations: ANAs, Anti-nuclear antibodies; AP, Autoimmune Psychosis; CASPR2, Contactin-associated protein-like 2; CSF, Cerebrospinal Fluid; CNS, Central Nervous System; EEG, Electroencephalography; FDG-PET, [^18^F] Fluorodeoxyglucose positron emission tomography; HC, Healthy Controls; IgG, Immunoglobulin G; LGI1, Leucine-rich glioma-inactivated 1; MOG, myelin oligodendrocyte glycoprotein; MRI, Magnetic resonance imaging; N, Number; NMDA-R, N-methyl-D-aspartate receptor; OCBs, oligoclonal bands; TPO, Thyroperoxidase; WBC, white blood cell; VGCCN, Voltage-gated calcium channel.

**Table 2:** Sociodemographic, psychometric, and neuropsychological characteristics of the study cohort of patients with suspected autoimmune psychosis (AP) spectrum syndromes and the healthy control (HC) group.

	**Patients (N=28)**^a^	**HC (N=28)**^a^	**p-value**^b^
**Sociodemographic and clinical findings**			

**Age in years**	38.1 ± 13.1	36.2 ± 11.4	p = 0.896
**Sex**			
female (%)	13 (46 %)	15 (54 %)	p = 0.593
male (%)	15 (54 %)	13 (46 %)	
**Body information**			
BMI (kg/m^2^)	25.7 ± 5.2	22.7 ± 2.4	**p = 0.002**
Handedness			p = 0.482
- Both (N = 23)	3 (13 %)	1 (3.6 %)	
- Left (N = 23)	2 (8.7 %)	3 (11 %)	
- Right (N = 23)	18 (78 %)	24 (86 %)	
**Mother tongue**			
German	27 (96 %)	26 (93 %)	p > 0.999
Chinese	0 (0 %)	1 (3.6 %)	
Rumanian	1 (3.6 %)	1 (3.6 %)	
**Academic degree**			
University degree	8 (29 %)	7 (25 %)	**p = 0.038**
High degree	8 (29 %)	18 (64 %)	
Middle degree	8 (29 %)	3 (11 %)	
Low degree	2 (7.1 %)	0 (0 %)	
Other qualification	1 (3.6 %)	0 (0 %)	
**Current employment status**			
Full-time job	2 (7.1 %)	14 (50 %)	**p < 0.001**
Part-time job	6 (21 %)	6 (21 %)	
Student	4 (14 %)	6 (21 %)	
Trainee	1 (3.6 %)	1 (3.6 %)	
Pensioner	8 (29 %)	0 (0 %)	
Unemployed	7 (25 %)	1 (3.6 %)	
**Current Psychiatric Medication (< 6 month)**			
Atypical Antipsychotics	11 (39 %)	0 (0 %)	–
Typical Antipsychotics	4 (14 %)	0 (0 %)	
Antidepressants	17 (61 %)	0 (0 %)	
Mood stabilizers	3 (11 %)	0 (0 %)	
Anticonvulsants	6 (21 %)	0 (0 %)	
Benzodiazepines	3 (11 %)	0 (0 %)	
**Psychometric and neuropsychological scores**			
	**p-value**^b^		**p-value**^b^

**PANSS**		**BDI-II** (N = 25/28)	**p < 0.001**
PANSS sum (N = 27/28)	**p < 0.001**	**EQ** (N = 25/28)	p = 0.824
PANSS positive (N = 27/28)	**p < 0.001**	**AQ** (N = 25/28)	**p = 0.002**
PANSS negative (N = 27/28)	**p < 0.001**	**WURS** (N = 23/28)	**p = 0.049**
		**ADHD-Checklist** (N = 22/28)	**p < 0.001**
		**STAI-G Trait** (N = 25/28)	**p < 0.001**
**ESI**		**TAP**	
ESI attention and speech impairment (N = 23/27)	**p = 0.007**	Alertness no warning tone (N = 20/27)	p = 0.096
ESI auditory uncertainty impairment (N = 23/27)	p = 0.054	Alertness warning tone	p = 0.114
ESI deviant perception impairment (N = 24/27)	**p = 0.043**	Phasic alertness (N = 20/27)	p = 0.281
ESI ideas of reference impairment (N = 23/27)	p = 0.062	Working memory mistakes (N = 20/27)	**p = 0.032**
ESI frankness impairment (N = 23/27)	p = 0.831	Working memory missings (N = 20/27)	**p = 0.004**
		Mental flexibility (N = 20/27)	**p < 0.001**
		Divided attention mistakes (N = 20/27)	p = 0.197
		Divided attention missings (N = 20/27)	**p = 0.009**
**VLMT**		**CFTR-20 IQ** (N = 19/24)	**p = 0.002**
VLMT Learning	**p < 0.001**	**SCL-90-R** (N = 24/28)	
VLMT False positive (N = 26/28)	p = 0.091	Hostility	**p = 0.002**
VLMT Preservations (N = 26/28)	p = 0.226	Anxiety	**p < 0.001**
VLMT Recognition (N = 26/28)	**p < 0.001**	Depression	**p < 0.001**
VLMT Consolidation	**p < 0.001**	Somatization	**p = 0.003**
		Obsessive-compulsive	**p < 0.001**
		Interpersonal sensitivity	**p = 0.002**
		Phobic anxiety	**p = 0.002**
		Paranoid ideation	**p = 0.009**
		Psychoticism	**p = 0.001**

Abbreviations: ADHD, Attention deficit hyperactivity disorder; AP, Autoimmune psychosis; AQ, Autism Spectrum Quotient; BDI-II, Beck Depression Inventory II; BMI, Body Mass Index; CFTR-20, Culture Fair Intelligence Testing; EQ, Cambridge Behaviour Scale-40; ESI, Eppendorfer Schizophrenia Inventory; HC, Healthy controls; PANSS, Positive and Negative Syndrome Scale; SCL-90R, Symptom Checklist-90-R; STAI-G, State-Trait Anxiety Inventory; SD, Standard deviation; TAP, Test of Attentional Performance; VLMT, Verbal Learning Memory Test; WURS, Wender Utah Rating Scale.

a Mean ± SD; n (%).

b Welch Two Sample *t*-test or Fisher's exact test or Wilcoxon rank sum test.

### 3.2 Differences in functional connectivity between patients and controls

**Fig. 2:**
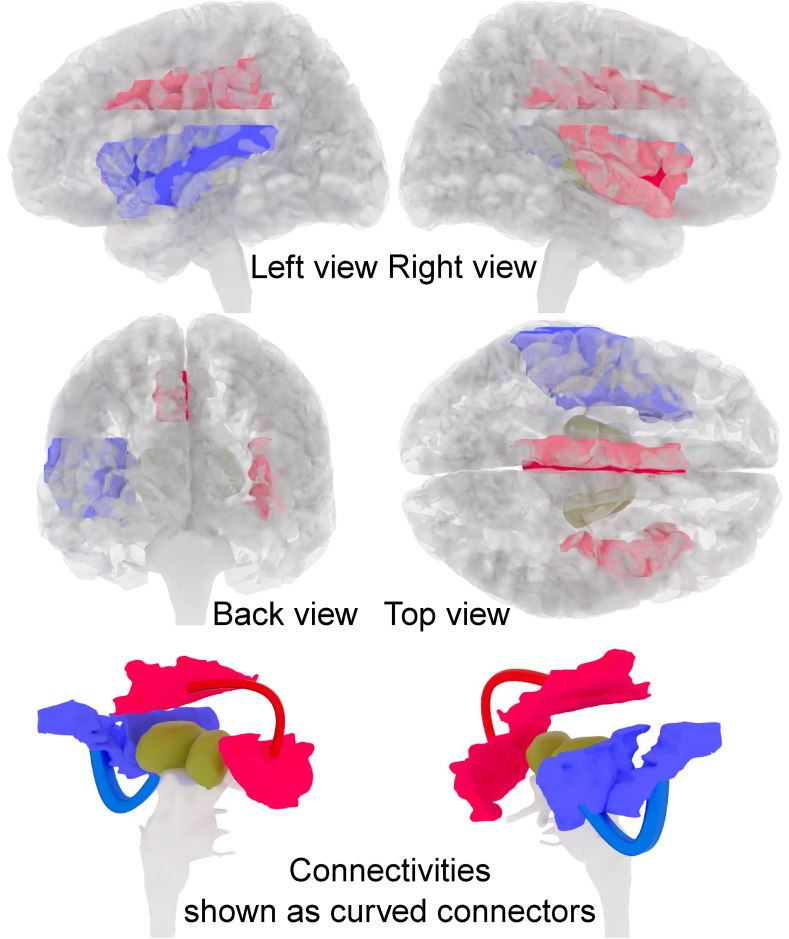
The main alterations in functional connectivity that remained significant after correction for multiple testing are shown. The functional connectivity between the left middle cingulate/paracingulate gyri and the right insula was identified to be significantly reduced in the suspected autoimmune psychosis spectrum syndrome compared to the healthy control group (marked in red). Increased connectivity between the left insula and the left superior temporal gyrus correlated with depressive symptoms measured via the Beck Depression Inventory (marked in blue). The bottom line visualizes these connectivities as curved connectors of corresponding color in perspective views without showing the cortical surface. (For interpretation of the references to color in this figure legend, the reader is referred to the Web version of this article.)

**Fig. 3:**
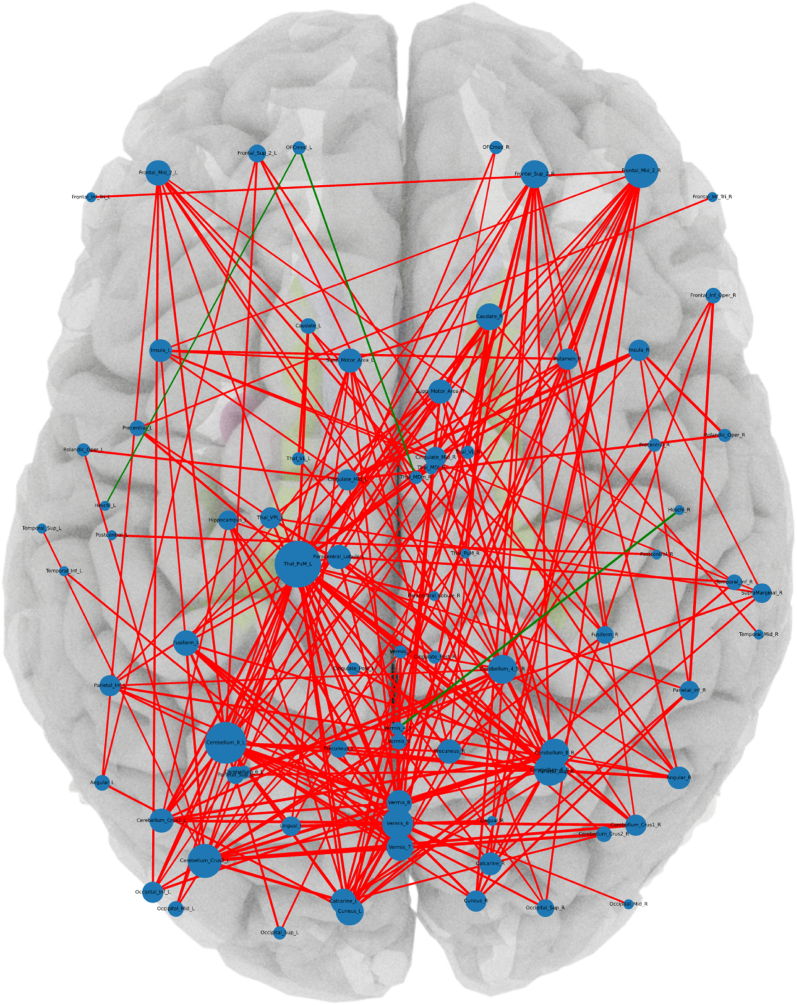
**Graphical representation of the altered functional connectivities with anatomical allocation (in the hypothesis-generating approach). The thickness of the connecting lines is shown according to the strength of the group effect. Red indicates reduced connectivity in the suspected autoimmune psychosis (AP) spectrum group (AP < HC), green indicates increased connectivity (AP > HC). The size of the hubs corresponds to the number of their significant connections. A lenient approach with a significance threshold of p < 0.01 was applied.** Abbreviations: ACC_sub, Anterior cingulate cortex, subgenual; ACC_sup, Anterior cingulate cortex, supracallosal; ACC_pre, Anterior cingulate cortex, pregenual; Amygdala, Amygdala; Angular, Angular gyrus; Calcarine, Calcarine fissure and surrounding cortex; Caudate, Caudate nucleus; Cerebellum_Crus1, Crus I of cerebellar hemisphere; Cerebellum_Crus2, Crus II of cerebellar hemisphere; Cerebellum_4_5, Lobule IV, V of cerebellar hemisphere; Cerebellum_6, Lobule VI of cerebellar hemisphere; Cerebellum_7b, Lobule VIIB of cerebellar hemisphere; Cerebellum_8, Lobule VIII of cerebellar hemisphere; Cerebellum_9, Lobule IX of cerebellar hemisphere; Cerebellum_10, Lobule X of cerebellar hemisphere; Cingulate_Ant, Anterior cingulate & paracingulate gyri; Cingulate_Mid, Middle cingulate & paracingulate gyri; Cingulate_Post, Posterior cingulate gyrus; Cuneus, Cuneus; Frontal_Inf_Oper, Inferior frontal gyrus, opercular part; Frontal_Inf_Orb, IFG pars orbitalis; Frontal_Inf_Tri, Inferior frontal gyrus, triangular part; Frontal_Med_Orb, Superior frontal gyrus, medial orbital; Frontal_Mid, Middle frontal gyrus; Frontal_Sup, Superior frontal gyrus, dorsolateral; Frontal_Sup_Med, Superior frontal gyrus, medial; Fusiform, Fusiform gyrus; Heschl, Heschl's gyrus; Hippocampus, Hippocampus; Insula, Insula; L, left; LC, Locus coeruleus; Lingual, Lingual gyrus; N_Acc, Nucleus accumbens; Occipital_Inf, Inferior occipital gyrus; Occipital_Mid, Middle occipital gyrus; Occipital_Sup, Superior occipital gyrus; OFCant, Anterior orbital gyrus; OFClat, Lateral orbital gyrus; OFCmed, Medial orbital gyrus; OFCpost, Posterior orbital gyrus; Olfactory, Olfactory cortex; Pallidum, Lenticular nucleus, Pallidum; Paracentral_Lobule, Paracentral lobule; ParaHippocampal, Parahippocampal gyrus; Parietal_Inf, Inferior parietal gyrus, excluding supramarginal and angular gyri; Parietal_Sup, Superior parietal gyrus; Precentral, Precentral gyrus; Precuneus, Precuneus; Postcentral, Postcentral gyrus; Putamen, Lenticular nucleus, Putamen; R, right; Raphe_D, Raphe nucleus, dorsal; Raphe_M, Raphe nucleus, median; Rectus, Gyrus rectus; Red_N, Red nucleus; Rolandic_Oper, Rolandic operculum; SN_pc, Substantia nigra, pars compacta; SN_pr, Substantia nigra, pars reticulata; Supramarginal, Supramarginal gyrus; Supp_Motor_Area, Supplementary motor area; Temporal_Inf, Inferior temporal gyrus; Temporal_Mid, Middle temporal gyrus; Temporal_Sup, Superior temporal gyrus; Temporal_Pole_Mid, Temporal pole: middle temporal gyrus; Temporal_Pole_Sup, Temporal pole: superior temporal gyrus; Thal, Thalamus; Thal_AV, Thalamus, Anteroventral Nucleus; Thal_IL, Intralaminar; Thal_LGN, Lateral geniculate; Thal_LP, Lateral posterior; Thal_MDl, Mediodorsal lateral parvocellular; Thal_MDm, Mediodorsal medial magnocellular; Thal_MGN, Medial Geniculate; Thal_PuA, Pulvinar anterior; Thal_PuL, Pulvinar lateral; Thal_PuM, Pulvinar medial; Thal_Re, Reuniens; Thal_VA, Ventral anterior; Thal_VL, Ventral lateral; Thal_VPL, Ventral posterolateral; Vermis_1_2, Lobule I, II of vermis; Vermis_3, Lobule III of vermis; Vermis_4_5, Lobule IV, V of vermis; Vermis_6, Lobule VI of vermis; Vermis_7, Lobule VII of vermis; Vermis_8, Lobule VIII of vermis; Vermis_9, Lobule IX of vermis; Vermis_10, Lobule X of vermis. (For interpretation of the references to color in this figure legend, the reader is referred to the Web version of this article.)

**Fig. 4:**
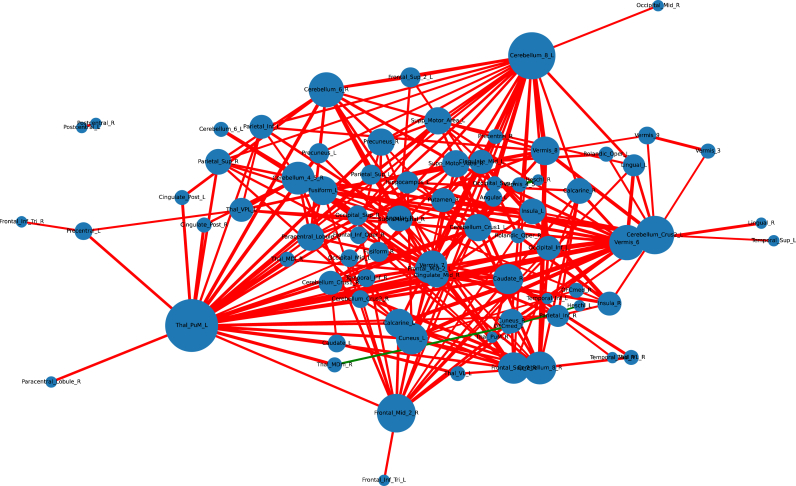
**Graphical representation of the altered functional connectivities with allocation to networks (in the hypothesis-generating approach). The thickness of the connecting lines is shown according to the strength of the group effect. Red indicates reduced connectivity in the suspected autoimmune psychosis (AP) spectrum syndrome group (AP < HC), green indicates increased connectivity (AP > HC). The size of the hubs corresponds to the number of their significant connections. A lenient approach with a significance threshold of p < 0.001 was applied.** Abbreviations: ACC_sub, Anterior cingulate cortex, subgenual; ACC_sup, Anterior cingulate cortex, supracallosal; ACC_pre, Anterior cingulate cortex, pregenual; Amygdala, Amygdala; Angular, Angular gyrus; Calcarine, Calcarine fissure and surrounding cortex; Caudate, Caudate nucleus; Cerebellum_Crus1, Crus I of cerebellar hemisphere; Cerebellum_Crus2, Crus II of cerebellar hemisphere; Cerebellum_4_5, Lobule IV, V of cerebellar hemisphere; Cerebellum_6, Lobule VI of cerebellar hemisphere; Cerebellum_7b, Lobule VIIB of cerebellar hemisphere; Cerebellum_8, Lobule VIII of cerebellar hemisphere; Cerebellum_9, Lobule IX of cerebellar hemisphere; Cerebellum_10, Lobule X of cerebellar hemisphere; Cingulate_Ant, Anterior cingulate & paracingulate gyri; Cingulate_Mid, Middle cingulate & paracingulate gyri; Cingulate_Post, Posterior cingulate gyrus; Cuneus, Cuneus; Frontal_Inf_Oper, Inferior frontal gyrus, opercular part; Frontal_Inf_Orb, IFG pars orbitalis; Frontal_Inf_Tri, Inferior frontal gyrus, triangular part; Frontal_Med_Orb, Superior frontal gyrus, medial orbital; Frontal_Mid, Middle frontal gyrus; Frontal_Sup, Superior frontal gyrus, dorsolateral; Frontal_Sup_Med, Superior frontal gyrus, medial; Fusiform, Fusiform gyrus; Heschl, Heschl's gyrus; Hippocampus, Hippocampus; Insula, Insula; L, left; LC, Locus coeruleus; Lingual, Lingual gyrus; N_Acc, Nucleus accumbens; Occipital_Inf, Inferior occipital gyrus; Occipital_Mid, Middle occipital gyrus; Occipital_Sup, Superior occipital gyrus; OFCant, Anterior orbital gyrus; OFClat, Lateral orbital gyrus; OFCmed, Medial orbital gyrus; OFCpost, Posterior orbital gyrus; Olfactory, Olfactory cortex; Pallidum, Lenticular nucleus, Pallidum; Paracentral_Lobule, Paracentral lobule; ParaHippocampal, Parahippocampal gyrus; Parietal_Inf, Inferior parietal gyrus, excluding supramarginal and angular gyri; Parietal_Sup, Superior parietal gyrus; Precentral, Precentral gyrus; Precuneus, Precuneus; Postcentral, Postcentral gyrus; Putamen, Lenticular nucleus, Putamen; R, right; Raphe_D, Raphe nucleus, dorsal; Raphe_M, Raphe nucleus, median; Rectus, Gyrus rectus; Red_N, Red nucleus; Rolandic_Oper, Rolandic operculum; SN_pc, Substantia nigra, pars compacta; SN_pr, Substantia nigra, pars reticulata; Supramarginal, Supramarginal gyrus; Supp_Motor_Area, Supplementary motor area; Temporal_Inf, Inferior temporal gyrus; Temporal_Mid, Middle temporal gyrus; Temporal_Sup, Superior temporal gyrus; Temporal_Pole_Mid, Temporal pole: middle temporal gyrus; Temporal_Pole_Sup, Temporal pole: superior temporal gyrus; Thal, Thalamus; Thal_AV, Thalamus, Anteroventral Nucleus; Thal_IL, Intralaminar; Thal_LGN, Lateral geniculate; Thal_LP, Lateral posterior; Thal_MDl, Mediodorsal lateral parvocellular; Thal_MDm, Mediodorsal medial magnocellular; Thal_MGN, Medial Geniculate; Thal_PuA, Pulvinar anterior; Thal_PuL, Pulvinar lateral; Thal_PuM, Pulvinar medial; Thal_Re, Reuniens; Thal_VA, Ventral anterior; Thal_VL, Ventral lateral; Thal_VPL, Ventral posterolateral; Vermis_1_2, Lobule I, II of vermis; Vermis_3, Lobule III of vermis; Vermis_4_5, Lobule IV, V of vermis; Vermis_6, Lobule VI of vermis; Vermis_7, Lobule VII of vermis; Vermis_8, Lobule VIII of vermis; Vermis_9, Lobule IX of vermis; Vermis_10, Lobule X of vermis. (For interpretation of the references to color in this figure legend, the reader is referred to the Web version of this article.)

***Connectivities****:* After correction for multiple testing, significantly reduced functional connectivity was observed between the right insula and the left middle cingulate/paracingulate gyri (p_adj_ = 0.025) in the patient compared to the HC group (Fig. 2). Exploratory analyses revealed widespread changes in functional connectivity between patients and HC participants across multiple brain regions. In total, 226 of the 5995 analyzed correlations (3.8 % of all region pairs) showed alterations in connectivity. The majority, more precisely 223/226 (99 %), were hypoconnectivities, while only 3/226 (1 %) were hyperconnectivities. The hypoconnectivities were widely distributed throughout the brain, whereas two of the three hyperconnectivities (67 %) involved frontal regions (Fig. 3, Fig. 4).

**Fig. 5:**
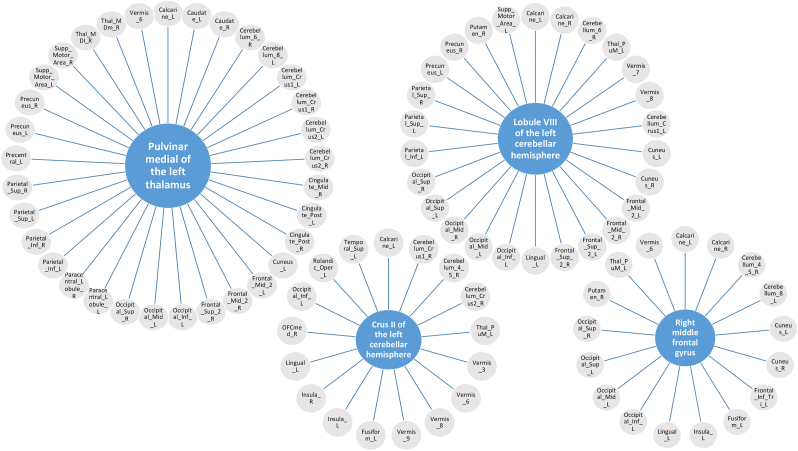
**Major hubs with functional dyconnectivities to 17–33 regions (in the hypothesis-generating approach). Hubs with functional dysconnectivities to 1 to 16 regions are not presented here (for overview see**Table 3). Abbreviations: Calcarine, Calcarine fissure and surrounding cortex; Caudate, Caudate nucleus; Cerebellum_Crus1, Crus I of cerebellar hemisphere; Cerebellum_Crus2, Crus II of cerebellar hemisphere; Cerebellum_4_5, Lobule IV, V of cerebellar hemisphere; Cerebellum_6, Lobule VI of cerebellar hemisphere; Cerebellum_8, Lobule VIII of cerebellar hemisphere; Cingulate_Mid, Middle cingulate & paracingulate gyri; Cingulate_Post, Posterior cingulate gyrus; Cuneus, Cuneus; Frontal_Inf_Tri, Inferior frontal gyrus, triangular part; Frontal_Mid, Middle frontal gyrus; Frontal_Sup, Superior frontal gyrus, dorsolateral; Fusiform, Fusiform gyrus; Insula, Insula; L, left; Lingual, Lingual gyrus; Occipital_Inf, Inferior occipital gyrus; Occipital_Mid, Middle occipital gyrus; Occipital_Sup, Superior occipital gyrus; OFCmed, Medial orbital gyrus; Paracentral_Lobule, Paracentral lobule; Parietal_Inf, Inferior parietal gyrus, excluding supramarginal and angular gyri; Parietal_Sup, Superior parietal gyrus; Precentral, Precentral gyrus; Precuneus, Precuneus; Putamen, Lenticular nucleus, Putamen; R, right; Rolandic_Oper, Rolandic operculum; Supp_Motor_Area, Supplementary motor area; Temporal_Sup, Superior temporal gyrus; Thal_MDl, Mediodorsal lateral parvocellular; Thal_MDm, Mediodorsal medial magnocellular; Thal_PuM, Pulvinar medial; Vermis_3, Lobule III of vermis; Vermis_6, Lobule VI of vermis; Vermis_7, Lobule VII of vermis; Vermis_8, Lobule VIII of vermis; Vermis_9, Lobule IX of vermis.

***Hubs:*** The most frequently altered connectivity hubs (uncorrected data with p < 0.01) are displayed in Fig. 5, with a comprehensive overview in Table 3. The medial pulvinar of the left thalamus was the most disconnected region, which exhibited functional dysconnectivity with 33 other regions. Overall, 79 of 170 analyzed regions (46 %) showed at least one altered functional connectivity. Of these 79 regions, 15 (19 %) were located in the cerebellum, 9 (11 %) in the frontal brain, 7 (9 %) in the thalami, 5 (6 %) in the occipital, and 4 (5 %) each in the parietal cortex, cuneus/precuneus, cingulate and paracingulate cortex, and temporal areas. In addition, 2 (3 %) each were identified in the caudate nucleus, calcarine fissure, insula, paracentral lobule, supplementary motor area, Rolandic opercula, Heschl's gyrus, angular gyrus, lingual gyrus, postcentral gyrus, and fusiform gyrus. Only 1 (1 %) was located in the left hippocampus, right putamen, and supramarginal gyrus, respectively.

**Table 3:** All hubs with functional dyconnectivities to 1–33 regions (in the hypothesis-generating approach). The four major hubs are summarized in Fig. 5.

Thal_PuM_L	33	Calcarine_L Caudate_L Caudate_R Cerebellum_6_R Cerebellum_8_L Cerebellum_Crus1_L Cerebellum_Crus1_R Cerebellum_Crus2_L Cerebellum_Crus2_R Cingulate_Mid_R Cingulate_Post_L Cingulate_Post_R Cuneus_L Frontal_Mid_2_L Frontal_Mid_2_R Frontal_Sup_2_R Occipital_Inf_L Occipital_Mid_L Occipital_Sup_R Paracentral_Lobule_L Paracentral_Lobule_R Parietal_Inf_L Parietal_Inf_R Parietal_Sup_L Parietal_Sup_R Precentral_L Precuneus_L Precuneus_R Supp_Motor_Area_L Supp_Motor_Area_R Thal_MDl_R Thal_MDm_R Vermis_6
Cerebellum_8_L	26	Calcarine_L Calcarine_R Cerebellum_6_R Thal_PuM_L Vermis_7 Vermis_8 Cerebellum_Crus1_L Cuneus_L Cuneus_R Frontal_Mid_2_L Frontal_Mid_2_R Frontal_Sup_2_L Frontal_Sup_2_R Lingual_L Occipital_Inf_L Occipital_Mid_L Occipital_Mid_R Occipital_Sup_L Occipital_Sup_R Parietal_Inf_L Parietal_Sup_L Parietal_Sup_R Precuneus_L Precuneus_R Putamen_R Supp_Motor_Area_L
Cerebellum_Crus2_L	17	Calcarine_L Cerebellum_Crus1_R Cerebellum_4_5_R Cerebellum_Crus2_R Thal_PuM_L Vermis_3 Vermis_6 Vermis_8 Vermis_9 Fusiform_L Insula_L Insula_R Lingual_L OFCmed_R Occipital_Inf_L Rolandic_Oper_L Temporal_Sup_L
Frontal_Mid_2_R	17	Calcarine_L Calcarine_R Cerebellum_4_5_R Cerebellum_8_L Cuneus_L Cuneus_R Frontal_Inf_Tri_L Fusiform_L Insula_L Lingual_L Occipital_Inf_L Occipital_Mid_L Occipital_Sup_L Occipital_Sup_R Putamen_R Thal_PuM_L Vermis_6
Cerebellum_6_R	14	Angular_R Cerebellum_8_L Thal_PuM_L Vermis_8 Cingulate_Post_L Frontal_Mid_2_L Frontal_Sup_2_L Frontal_Sup_2_R Paracentral_Lobule_L Parietal_Sup_L Parietal_Sup_R Precuneus_L Precuneus_R Supp_Motor_Area_R
Vermis_6	14	Calcarine_L Calcarine_R Cerebellum_Crus2_L Cuneus_L Cuneus_R Frontal_Mid_2_R Fusiform_L Hippocampus_L Lingual_L Lingual_R Supp_Motor_Area_L Supp_Motor_Area_R Thal_PuM_L Thal_VPL_L
Cerebellum_4_5_R	12	Angular_R Cerebellum_6_L Thal_VPL_L Cerebellum_Crus1_L Cerebellum_Crus2_L Cingulate_Mid_R Cingulate_Post_R Frontal_Mid_2_L Frontal_Mid_2_R Paracentral_Lobule_L Parietal_Inf_L Parietal_Sup_R
Cerebellum_8_R	12	Angular_R Calcarine_L Calcarine_R Thal_VL_L Thal_VL_R Vermis_8 Cerebellum_Crus1_L Cerebellum_Crus1_R Cuneus_L Cuneus_R Fusiform_L Occipital_Inf_L
Cuneus_L	11	Angular_L Cerebellum_8_L Cerebellum_8_R Temporal_Inf_R Thal_PuM_L Vermis_6 Vermis_7 Frontal_Mid_2_R Frontal_Sup_2_R Hippocampus_L OFCmed_R
Frontal_Sup_2_R	11	Angular_R Calcarine_L Calcarine_R Cerebellum_6_R Cerebellum_8_L Cuneus_L Cuneus_R Fusiform_L Fusiform_R Thal_PuM_L Vermis_7
Caudate_R	10	Temporal_Mid_R Thal_MDl_R Thal_PuM_L Thal_PuM_R Thal_VL_R Cingulate_Mid_R Paracentral_Lobule_L Precuneus_R Supp_Motor_Area_L Supp_Motor_Area_R
Vermis_7	10	Cerebellum_8_L Cuneus_L Frontal_Sup_2_R Fusiform_L Precentral_R Precuneus_L Precuneus_R Supp_Motor_Area_R Temporal_Inf_L Vermis_4_5
Calcarine_L	9	Cerebellum_8_L Cerebellum_8_R Cerebellum_Crus2_L Cerebellum_Crus2_R Parietal_Sup_R Thal_PuM_L Vermis_6 Frontal_Mid_2_R Frontal_Sup_2_R
Vermis_8	9	Cerebellum_6_R Cerebellum_8_L Cerebellum_8_R Cerebellum_Crus1_L Cerebellum_Crus2_L Fusiform_L Paracentral_Lobule_L Parietal_Inf_L Precentral_R
Frontal_Mid_2_L	9	Angular_L Angular_R Cerebellum_4_5_R Cerebellum_6_R Cerebellum_8_L Fusiform_R Occipital_Inf_L Temporal_Inf_R Thal_PuM_L
Fusiform_L	9	Frontal_Mid_2_R Frontal_Sup_2_R Cerebellum_6_L Cerebellum_8_R Cerebellum_Crus2_L Paracentral_Lobule_L Vermis_6 Vermis_7 Vermis_8
Cerebellum_Crus1_L	8	Cerebellum_4_5_R Cerebellum_8_L Cerebellum_8_R Cerebellum_Crus1_R Thal_PuM_L Vermis_8 Hippocampus_L Supp_Motor_Area_R
Precuneus_R	8	Frontal_Inf_Oper_R Fusiform_R Caudate_R Cerebellum_6_R Cerebellum_8_L Thal_PuM_L Vermis_7 Supp_Motor_Area_L
Paracentral_Lobule_L	8	Fusiform_L Hippocampus_L Caudate_R Cerebellum_4_5_R Cerebellum_6_R Temporal_Inf_R Thal_PuM_L Vermis_8
Supp_Motor_Area_L	8	Caudate_R Cerebellum_8_L Insula_L Lingual_L Occipital_Sup_R Precuneus_R Thal_PuM_L Vermis_6
Supp_Motor_Area_R	8	Caudate_R Cerebellum_6_R Cerebellum_Crus1_L Fusiform_R Thal_PuM_L Thal_VPL_L Vermis_6 Vermis_7
Angular_R	7	Cerebellum_4_5_R Cerebellum_6_R Cerebellum_8_R Frontal_Mid_2_L Frontal_Sup_2_L Frontal_Sup_2_R Parietal_Inf_L
Parietal_Sup_R	7	Calcarine_L Frontal_Inf_Oper_R Cerebellum_4_5_R Cerebellum_6_R Cerebellum_8_L SupraMarginal_R Thal_PuM_L
Calcarine_R	7	Cerebellum_8_L Cerebellum_8_R Parietal_Inf_R Vermis_6 Frontal_Mid_2_R Frontal_Sup_2_R Hippocampus_L
Insula_L	7	Frontal_Mid_2_R Cerebellum_Crus2_L Cingulate_Mid_L Cingulate_Mid_R Putamen_R SupraMarginal_R Supp_Motor_Area_L
Cerebellum_Crus1_R	6	Caudate_L Cerebellum_Crus1_L Cerebellum_8_R Cerebellum_Crus2_L Thal_PuM_L Putamen_R
Thal_VPL_L	6	Cerebellum_4_5_R Parietal_Inf_L Precentral_L Supp_Motor_Area_R SupraMarginal_R Vermis_6
Cingulate_Mid_L	6	Putamen_R Vermis_9 Insula_L Insula_R Rolandic_Oper_L Rolandic_Oper_R
Putamen_R	6	Cingulate_Mid_L Cingulate_Mid_R Frontal_Mid_2_R Insula_L Cerebellum_8_L Cerebellum_Crus1_R
Cingulate_Mid_R	6	Caudate_R Cerebellum_4_5_R Putamen_R Thal_PuM_L Insula_L Insula_R
Cuneus_R	6	Cerebellum_8_L Cerebellum_8_R Parietal_Inf_R Vermis_6 Frontal_Mid_2_R Frontal_Sup_2_R
Occipital_Inf_L	6	Frontal_Mid_2_L Frontal_Mid_2_R Cerebellum_8_L Cerebellum_8_R Cerebellum_Crus2_L Thal_PuM_L
Insula_R	6	Cerebellum_Crus2_L Cingulate_Mid_L Cingulate_Mid_R Parietal_Inf_R SupraMarginal_R Rolandic_Oper_R
Parietal_Inf_L	6	Angular_R Cerebellum_4_5_R Cerebellum_8_L Thal_PuM_L Thal_VPL_L Vermis_8
Parietal_Inf_R	5	Calcarine_R Cuneus_R Frontal_Inf_Oper_R Insula_R Thal_PuM_L
Lingual_L	5	Frontal_Mid_2_R Cerebellum_8_L Cerebellum_Crus2_L Vermis_6 Supp_Motor_Area_L
Hippocampus_L	5	Calcarine_R Cerebellum_Crus1_L Cuneus_L Paracentral_Lobule_L Vermis_6
SupraMarginal_R	5	Insula_L Insula_R Parietal_Sup_L Parietal_Sup_R Thal_VPL_L
Fusiform_R	4	Frontal_Mid_2_L Frontal_Sup_2_R Precuneus_R Supp_Motor_Area_R
Occipital_Sup_R	4	Frontal_Mid_2_R Cerebellum_8_L Thal_PuM_L Supp_Motor_Area_L
Frontal_Sup_2_L	4	Angular_L Angular_R Cerebellum_6_R Cerebellum_8_L
Parietal_Sup_L	4	Cerebellum_6_R Cerebellum_8_L SupraMarginal_R Thal_PuM_L
Precuneus_L	4	Cerebellum_6_R Cerebellum_8_L Thal_PuM_L Vermis_7
Cerebellum_Crus2_R	3	Calcarine_L Cerebellum_Crus2_L Thal_PuM_L
Caudate_L	3	Cerebellum_Crus1_R Thal_PuM_L Thal_VL_L
Vermis_9	3	Cerebellum_Crus2_L Cingulate_Mid_L Vermis_3
Angular_L	3	Cuneus_L Frontal_Mid_2_L Frontal_Sup_2_L
Temporal_Inf_R	3	Cuneus_L Frontal_Mid_2_L Paracentral_Lobule_L
Frontal_Inf_Oper_R	3	Parietal_Inf_R Parietal_Sup_R Precuneus_R
Occipital_Mid_L	3	Frontal_Mid_2_R Cerebellum_8_L Thal_PuM_L
Precentral_L	3	Frontal_Inf_Tri_R Thal_PuM_L Thal_VPL_L
Thal_VL_L	2	Caudate_L Cerebellum_8_R
Thal_MDl_R	2	Caudate_R Thal_PuM_L
Thal_VL_R	2	Caudate_R Cerebellum_8_R
Cerebellum_6_L	2	Cerebellum_4_5_R Fusiform_L
Vermis_3	2	Cerebellum_Crus2_L Vermis_9
Cingulate_Post_L	2	Cerebellum_6_R Thal_PuM_L
Cingulate_Post_R	2	Cerebellum_4_5_R Thal_PuM_L
Occipital_Sup_L	2	Frontal_Mid_2_R Cerebellum_8_L
Vermis_4_5	2	Heschl_R Vermis_7
OFCmed_L	2	Heschl_L Thal_MDm_R
Thal_MDm_R	2	OFCmed_L Thal_PuM_L
OFCmed_R	2	Cerebellum_Crus2_L Cuneus_L
Precentral_R	2	Vermis_7 Vermis_8
Rolandic_Oper_L	2	Cerebellum_Crus2_L Cingulate_Mid_L
Rolandic_Oper_R	2	Cingulate_Mid_L Insula_R
Temporal_Mid_R	1	Caudate_R
Thal_PuM_R	1	Caudate_R
Frontal_Inf_Tri_L	1	Frontal_Mid_2_R
Heschl_R	1	Vermis_4_5
Lingual_R	1	Vermis_6
Heschl_L	1	OFCmed_L
Occipital_Mid_R	1	Cerebellum_8_L
Paracentral_Lobule_R	1	Thal_PuM_L
Postcentral_L	1	Postcentral_R
Postcentral_R	1	Postcentral_L
Frontal_Inf_Tri_R	1	Precentral_L
Temporal_Inf_L	1	Vermis_7
Temporal_Sup_L	1	Cerebellum_Crus2_L

Abbreviations: ACC_sub, Anterior cingulate cortex, subgenual; ACC_sup, Anterior cingulate cortex, supracallosal; ACC_pre, Anterior cingulate cortex, pregenual; Amygdala, Amygdala; Angular, Angular gyrus; Calcarine, Calcarine fissure and surrounding cortex; Caudate, Caudate nucleus; Cerebellum_Crus1, Crus I of cerebellar hemisphere; Cerebellum_Crus2, Crus II of cerebellar hemisphere; Cerebellum_4_5, Lobule IV, V of cerebellar hemisphere; Cerebellum_6, Lobule VI of cerebellar hemisphere; Cerebellum_7b, Lobule VIIB of cerebellar hemisphere; Cerebellum_8, Lobule VIII of cerebellar hemisphere; Cerebellum_9, Lobule IX of cerebellar hemisphere; Cerebellum_10, Lobule X of cerebellar hemisphere; Cingulate_Ant, Anterior cingulate & paracingulate gyri; Cingulate_Mid, Middle cingulate & paracingulate gyri; Cingulate_Post, Posterior cingulate gyrus; Cuneus, Cuneus; Frontal_Inf_Oper, Inferior frontal gyrus, opercular part; Frontal_Inf_Orb, IFG pars orbitalis; Frontal_Inf_Tri, Inferior frontal gyrus, triangular part; Frontal_Med_Orb, Superior frontal gyrus, medial orbital; Frontal_Mid, Middle frontal gyrus; Frontal_Sup, Superior frontal gyrus, dorsolateral; Frontal_Sup_Med, Superior frontal gyrus, medial; Fusiform, Fusiform gyrus; Heschl, Heschl's gyrus; Hippocampus, Hippocampus; Insula, Insula; L, left; LC, Locus coeruleus; Lingual, Lingual gyrus; N_Acc, Nucleus accumbens; Occipital_Inf, Inferior occipital gyrus; Occipital_Mid, Middle occipital gyrus; Occipital_Sup, Superior occipital gyrus; OFCant, Anterior orbital gyrus; OFClat, Lateral orbital gyrus; OFCmed, Medial orbital gyrus; OFCpost, Posterior orbital gyrus; Olfactory, Olfactory cortex; Pallidum, Lenticular nucleus, Pallidum; Paracentral_Lobule, Paracentral lobule; ParaHippocampal, Parahippocampal gyrus; Parietal_Inf, Inferior parietal gyrus, excluding supramarginal and angular gyri; Parietal_Sup, Superior parietal gyrus; Precentral, Precentral gyrus; Precuneus, Precuneus; Postcentral, Postcentral gyrus; Putamen, Lenticular nucleus, Putamen; R, right; Raphe_D, Raphe nucleus, dorsal; Raphe_M, Raphe nucleus, median; Rectus, Gyrus rectus; Red_N, Red nucleus; Rolandic_Oper, Rolandic operculum; SN_pc, Substantia nigra, pars compacta; SN_pr, Substantia nigra, pars reticulata; Supramarginal, Supramarginal gyrus; Supp_Motor_Area, Supplementary motor area; Temporal_Inf, Inferior temporal gyrus; Temporal_Mid, Middle temporal gyrus; Temporal_Sup, Superior temporal gyrus; Temporal_Pole_Mid, Temporal pole: middle temporal gyrus; Temporal_Pole_Sup, Temporal pole: superior temporal gyrus; Thal, Thalamus; Thal_AV, Thalamus, Anteroventral Nucleus; Thal_IL, Intralaminar; Thal_LGN, Lateral geniculate; Thal_LP, Lateral posterior; Thal_MDl, Mediodorsal lateral parvocellular; Thal_MDm, Mediodorsal medial magnocellular; Thal_MGN, Medial Geniculate; Thal_PuA, Pulvinar anterior; Thal_PuL, Pulvinar lateral; Thal_PuM, Pulvinar medial; Thal_Re, Reuniens; Thal_VA, Ventral anterior; Thal_VL, Ventral lateral; Thal_VPL, Ventral posterolateral; Vermis_1_2, Lobule I, II of vermis; Vermis_3, Lobule III of vermis; Vermis_4_5, Lobule IV, V of vermis; Vermis_6, Lobule VI of vermis; Vermis_7, Lobule VII of vermis; Vermis_8, Lobule VIII of vermis; Vermis_9, Lobule IX of vermis; Vermis_10, Lobule X of vermis.

### 3.3 Correlations with psychometric, EEG, and CSF findings in the patient group

**Fig. 6:**
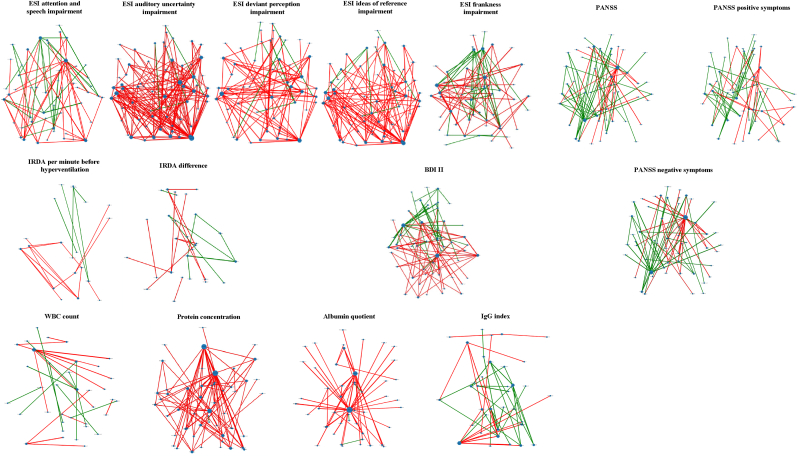
**Graphical presentation of the influence of selected psychometric scores, electroencephalography parameters (IRDAs/IRTAs), and cerebrospinal fluid finding on the correlation between two regions of interest for all combinations in the suspected AP spectrum syndrome group (in the hypothesis-generating approach). Linear models were calculated independently for each scale and the threshold of p < 0.01 was used. In red, correlations with hypoconnectivities are presented. In green, correlations with hyperconnectivites are shown.** Abbreviations: ACC_sub, Anterior cingulate cortex, subgenual; ACC_sup, Anterior cingulate cortex, supracallosal; ACC_pre, Anterior cingulate cortex, pregenual; Amygdala, Amygdala; Angular, Angular gyrus; BDI-II, Beck Depression Inventory II; Calcarine, Calcarine fissure and surrounding cortex; Caudate, Caudate nucleus; Cerebellum_Crus1, Crus I of cerebellar hemisphere; Cerebellum_Crus2, Crus II of cerebellar hemisphere; Cerebellum_4_5, Lobule IV, V of cerebellar hemisphere; Cerebellum_6, Lobule VI of cerebellar hemisphere; Cerebellum_7b, Lobule VIIB of cerebellar hemisphere; Cerebellum_8, Lobule VIII of cerebellar hemisphere; Cerebellum_9, Lobule IX of cerebellar hemisphere; Cerebellum_10, Lobule X of cerebellar hemisphere; Cingulate_Ant, Anterior cingulate & paracingulate gyri; Cingulate_Mid, Middle cingulate & paracingulate gyri; Cingulate_Post, Posterior cingulate gyrus; Cuneus, Cuneus; ESI, Eppendorfer Schizophrenia Inventory; Frontal_Inf_Oper, Inferior frontal gyrus, opercular part; Frontal_Inf_Orb, IFG pars orbitalis; Frontal_Inf_Tri, Inferior frontal gyrus, triangular part; Frontal_Med_Orb, Superior frontal gyrus, medial orbital; Frontal_Mid, Middle frontal gyrus; Frontal_Sup, Superior frontal gyrus, dorsolateral; Frontal_Sup_Med, Superior frontal gyrus, medial; Fusiform, Fusiform gyrus; Heschl, Heschl's gyrus; Hippocampus, Hippocampus; IgG index, immunoglobulin G index; Insula, Insula; L, IRDA, intermittent rhythmic delta/theta activity; left; LC, Locus coeruleus; Lingual, Lingual gyrus; N_Acc, Nucleus accumbens; Occipital_Inf, Inferior occipital gyrus; Occipital_Mid, Middle occipital gyrus; Occipital_Sup, Superior occipital gyrus; OFCant, Anterior orbital gyrus; OFClat, Lateral orbital gyrus; OFCmed, Medial orbital gyrus; OFCpost, Posterior orbital gyrus; Olfactory, Olfactory cortex; Pallidum, Lenticular nucleus, Pallidum; PANSS, Positive and Negative Syndrome Scale; Paracentral_Lobule, Paracentral lobule; ParaHippocampal, Parahippocampal gyrus; Parietal_Inf, Inferior parietal gyrus, excluding supramarginal and angular gyri; Parietal_Sup, Superior parietal gyrus; Precentral, Precentral gyrus; Precuneus, Precuneus; Postcentral, Postcentral gyrus; Putamen, Lenticular nucleus, Putamen; R, right; Raphe_D, Raphe nucleus, dorsal; Raphe_M, Raphe nucleus, median; Rectus, Gyrus rectus; Red_N, Red nucleus; Rolandic_Oper, Rolandic operculum; SN_pc, Substantia nigra, pars compacta; SN_pr, Substantia nigra, pars reticulata; Supramarginal, Supramarginal gyrus; Supp_Motor_Area, Supplementary motor area; Temporal_Inf, Inferior temporal gyrus; Temporal_Mid, Middle temporal gyrus; Temporal_Sup, Superior temporal gyrus; Temporal_Pole_Mid, Temporal pole: middle temporal gyrus; Temporal_Pole_Sup, Temporal pole: superior temporal gyrus; Thal, Thalamus; Thal_AV, Thalamus, Anteroventral Nucleus; Thal_IL, Intralaminar; Thal_LGN, Lateral geniculate; Thal_LP, Lateral posterior; Thal_MDl, Mediodorsal lateral parvocellular; Thal_MDm, Mediodorsal medial magnocellular; Thal_MGN, Medial Geniculate; Thal_PuA, Pulvinar anterior; Thal_PuL, Pulvinar lateral; Thal_PuM, Pulvinar medial; Thal_Re, Reuniens; Thal_VA, Ventral anterior; Thal_VL, Ventral lateral; Thal_VPL, Ventral posterolateral; Vermis_1_2, Lobule I, II of vermis; Vermis_3, Lobule III of vermis; Vermis_4_5, Lobule IV, V of vermis; Vermis_6, Lobule VI of vermis; Vermis_7, Lobule VII of vermis; Vermis_8, Lobule VIII of vermis; Vermis_9, Lobule IX of vermis; Vermis_10, Lobule X of vermis; WBC, white blood cell. (For interpretation of the references to color in this figure legend, the reader is referred to the Web version of this article.)

Fig. 6 provides a graphical summary with detailed data in the Supplementary Tables.

***Connectivities:*** After correction for multiple comparisons, increased connectivity between the left insula and the left superior temporal gyrus correlated significantly with the BDI-II sum score (p_adj_ = 0.043; Fig. 2). Exploratory correlations identified a predominantly global, significantly reduced functional connectivity correlating with the ESI scores. The number of correlations between functional connectivity and ESI subscores was as follows: attention and speech subscore: 37 (0.62 %) reduced connectivities and 24 (0.40 %) increased connectivities; auditory uncertainty: 166 (2.8 %) decreased and 4 (0.07 %) increased; deviant perception impairment: 86 (1.43 %) decreased and 6 (0.10 %) increased; ideas of reference: 103 (1.71 %) decreased and 7 (0.12 %) increased; and frankness impairment: 43 (0.72 %) decreased and 28 (0.47 %) increased. There were more positive correlations between functional connectivity and the PANSS for the negative, positive, and sum scores: PANSS negative score: 26 (0.43 %) decreased and 30 (0.50 %) increased; PANSS positive score: 21 (0.35 %) decreased and 28 (0.47 %) increased; and PANSS sum score: 25 (0.42 %) decreased and 36 (0.60 %) increased. Overall, 78 (1.30 %) correlations showed significantly altered functional connectivity associated with the BDI-II, of which 48 (0.80 %) indicated decreased connectivity and 30 (0.50 %) showed increased connectivity. EEG analyses showed that IRDA/IRTA rates before HV were associated with 14 (0.23 %) decreased and 5 (0.08 %) increased connectivities, while the IRDA/IRTA difference presented 13 (0.22 %) decreased and 8 (0.13 %) increased connectivities. Further correlations between functional connectivity and CSF values yielded the following results: The protein values and AQ were associated with globally decreased functional connectivity, while the IgG index showed more positive correlations [WBC count: 15 (0.25 %) decreased and 11 (0.18 %) increased; protein levels: 82 (1.37 %) decreased and 1 (0.02 %) increased; AQ: 50 (0.83 %) decreased and 3 (0.05 %) increased; and IgG index: 16 (0.27 %) decreased and 26 (0.43 %) increased].

***Hubs:*** The 170 regions analyzed that exhibited at least one altered connectivity, were further examined for exploratory correlations with psychometric and clinical scores. Several altered connectivities were associated with ESI subscores: attention and speech subscore: 55 (32 %) hubs involved; auditory uncertainty: 71 (42 %) hubs involved; deviant perception impairment: 58 (34 %) hubs involved; ideas of reference: 64 (38 %) hubs involved; and frankness impairment: 54 (32 %) hubs involved. Additional associations were found with the PANSS and BDI-II: PANSS (PANSS negative score: 55 (32 %) hubs involved; PANSS positive score: 53 (31 %) hubs involved; PANSS sum scores: 58 (34 %) hubs involved); and BDI-II (58 (34 %) hubs involved). Clinical data on EEG with pre-HV IRDA/IRTA rates showed involvement of 21 (12 %), while IRDA/IRTA difference involved 28 (17 %) hubs. CSF findings correlated with the following number of hubs (each showing at least one altered connectivity): WBC count: 36 (21 %), protein levels: 54 (32 %), AQ: 50 (29 %), and IgG index: 39 (23 %).

***Descriptive report of the correlations:*** Diverse and extensive alterations in ESI subscores were observed, generally reflecting reduced functional connectivity. Higher PANSS negative and sum scores were associated with increased functional connectivity. Notably, the left cerebellum exhibited increased connectivity in association with PANSS scores, particularly with frontal regions. Although higher BDI-II scores were largely associated with decreased connectivity, some frontal connections – many involving the left insula – were enhanced. IRDAs/IRTA before HV and IRDA/IRTA differences showed relatively fewer correlations with functional connectivity, but when present, increased functional connectivity was primarily observed in frontal and temporal regions of the right hemisphere. CSF parameters showed a more heterogeneous pattern: WBC and the IgG index were each linked to both decreased and increased connectivity, while protein levels and AQ were predominantly associated with reduced connectivity, particularly in cerebellar regions.

## 4 Discussion

This study was the first to address the question of how suspected autoimmune causality in patients with primary psychiatric symptoms affects functional brain connectivity. Especially, the functional connectivity between the left middle cingulate/paracingulate gyri and the right insula was strongly reduced in patients with suspected AP spectrum syndromes. The further results indicate a global decrease in functional connectivity affecting 3.8 % of all connectivities in patients – only few, mainly frontal connections, showed increased functional connectivity. The most disconnected hub was the medial pulvinar of the left thalamus. By employing a lenient (exploratory) threshold, 46 % of all brain regions displayed at least one altered connectivity, with the most frequent changes in the cerebellum, frontal regions, and both thalami. In addition, increased connectivity between the left insula and the left superior temporal gyrus was linked to more severe depressive symptoms.

***Previous studies*** with comparable case-control designs in AP/APS patients have not been published, as shown by our PubMed search. Nonetheless, studies in neurological patient groups investigating AE cases have consistently demonstrated functional connectivity abnormalities, even beyond structurally altered regions (Peer et al., 2017; Heine et al., 2018; Li et al., 2021; Hartung et al., 2024). For instance, Peer et al. (2017) reported extensive alterations in functional connectivity in 43 patients with NMDA-R encephalitis (72 % had normal clinical MRI), including hippocampal, medial temporal, default mode network, and frontotemporal connections. In addition, various large-scale networks, including sensorimotor, frontoparietal, lateral temporal, and visual networks, showed disturbed functional connectivity. Another research group examined 22 patients with NMDA-R encephalitis and 22 HCs, identifying a major hub with reduced connectivity in the left insula through nodal clustering coefficient and nodal local efficiency calculations (Li et al., 2021). Heine and colleagues (2018) reported altered network connectivity in 21 patients with LGI1 encephalitis (78 % had pathological clinical MRI), particularly within the default mode network and hippocampus, as well as within the sensorimotor, salience, and higher visual networks, which was visually the most affected region. Notably, insular hyperconnectivity with the salience and default mode networks was associated with memory impairment. Unlike our study, these investigations focused on more homogenous neurological patient groups with similar neuronal antibodies, primarily exhibiting neurological symptoms such as epileptic seizures, often accompanied by psychiatric symptoms.

***From a clinical and pathophysiological perspective***, the current study suggests that antibody-mediated processes significantly affect functional connectivity, even in psychiatric patients where autoimmune causes are suspected. In the same patient group (albeit slightly different subgroups), morphometric data showed only mild, global reductions in gray matter volumes and secondary increased CSF volume (von Zedtwitz et al., 2025b), whereas no neurometabolic group-level abnormalities were detected with MRSI (Endres et al., 2025). In contrast, functional connectivity analyses revealed more pronounced group differences. The study's key findings highlight reduced functional connectivity between the left middle cingulate/paracingulate gyri and the right insula in the patient group. In addition, increased connectivity between the left insula and the left superior temporal gyrus correlated with the severity of depressive symptoms. Depressive symptoms were prevalent in this cohort, where affective symptoms were present in the majority of cases. From a functional connectivity perspective, these findings suggest that some functional hypoconnectivites in patients with suspected AP spectrum syndromes might differentiate them from controls, but that there could still a relationship between functional hyperconnectivity and some (especially depressive) symptoms in the patient group, which might represent secondary compensatory processes. From the view of localization, the insula — which is known to be relevant for emotional, introspective and cognitive processes and a major disconnected hub (on both sides) in this patient cohort (see Table 3) — has previously been identified as a disrupted hub in studies on neurological patients with NMDA-R encephalitis (Li et al., 2021). In LGI1 encephalitis, insular hyperconnectivities has been associated with memory deficits (Heine et al., 2018). Furthermore, the insula is one of the most commonly affected regions in primary psychotic disorders, given its role in emotional, cognitive, and somatosensory processes (Kittleson et al., 2024). The alterations in the functional connectivity of the insula could therefore explain (at least partly) the link between autoimmunity and psychotic/affective symptoms. Exploratory analyses revealed a global cluster of altered connectivities (in 3.8 % of all connectivities), with hypoconnectivities accounting for 99 %. Similar global connectivity changes have also been observed in NMDA-R encephalitis (Peer et al., 2017). Apparently, antibody-mediated processes may induce widespread dysfunction largely unrecognized by structural (von Zedtwitz et al., 2025b) or neurochemical imaging (Endres et al., 2025), which could account for the severity and complexity of these clinical presentations despite largely normal conventional MRI findings. This patient cohort showed considerable neurocognitive deficits, and their presentations were not limited to purely schizophreniform syndromes—as often observed in AP/APS, where clinical manifestations often extend beyond classical psychosis (Endres et al., 2020a, 2022a, 2022b; Tebartz van Elst et al., 2025). Consistent with the AP consensus criteria, no uniform clinical syndrome is required (Pollak et al., 2020). This aligns with evidence that CNS antibody binding is often multifocal, extending beyond the hippocampus, as demonstrated by binding patterns on rat brain slices (Dalmau et al., 2017). In this patient group, antibodies against blood vessels predominated, typically presenting throughout the brain's vasculature and possibly causing a global effect on brain connectivity (Endres et al., 2022c). In terms of specific regions, the medial pulvinar nucleus of the left thalamus was the most disconnected hub, a finding that aligns with the pulvinar's extensive cortical connections and role in bidirectional communication (Cortes et al., 2024). Severe dysconnectivity in this critical relay station may impair cognitive integration (Cortes et al., 2024). The most frequent alterations occurred in the 1) cerebellum, 2) the frontal brain, and 3) both thalami. The strong cerebellar involvement underscores its essential role in higher mental processes, as described by Schmahmann and colleagues in their concept of the “cerebellar cognitive affective syndrome” (Schmahmann et al., 1998; Schmahmann, 2019). Similarly, Ito proposed the cerebellum as an “internal modeling machine”, suggesting that it modulates psychological functions in a manner analogous to motor functions (Ito, 2006, 2008). It is also notable that most CNS antibodies target the cerebellum, where most antigens are highly expressed (Dalmau et al., 2017). Additional antibodies, such as the Yo antibodies (positive in one patient of this cohort), are typically associated with autoimmune cerebellitis (Dalmau et al., 2017; Dalmau and Graus, 2018). Frontal involvement is also well documented in psychosis, particularly in frontotemporal circuits (see Gur et al., 2007). The exploratory analyses of associations — which should be regarded as preliminary findings and have to be replicated in further confirmatory studies — further revealed that higher ESI subscores were generally associated with reduced functional connectivity, whereas higher PANSS scores were predominantly associated with increased connectivity. This divergence suggests that certain psychotic symptoms may be associated with hyperconnectivity, while others may correspond to hypoconnectivity. IRDA/IRTA before HV and IRDA/IRTA-difference predominantly correlated with fewer functional connectivities, although those observed were primarily localized in right frontal and temporal regions. This finding could indicate a frontotemporal origin of IRDA/IRTA. Interestingly, CSF protein levels and the AQ — two indicators of blood-brain barrier integrity — had stronger associations with reduced functional connectivity than did inflammatory CSF markers, raising the possibility that even “non-specific” parameters might contribute to hypoconnectivity.

***From a methodological perspective***, a relatively lenient threshold of p < 0.01 for initial hypothesis generation was adopted. However, results that survived multiple tests were also reported. The MREG/fMRI protocol allowed ultrafast acquisition (TR = 100 ms) and thus a higher temporal resolution for controlling cardiac and respiratory confounders (Hennig et al., 2021). While this approach is technologically advanced, it produces very large datasets and raises questions about feasibility in routine clinical practice. Future research should clarify whether such high temporal resolution is indispensable.

***In terms of limitations***, not all patients from the broader project (von Zedtwitz et al., 2025b) could be included in the MREG analysis, largely owing to incomplete physiologic recordings (respiration and ECG). Datasets without valid physiologic data were excluded, leaving 28 matched patient–control pairs with high-quality data. This sample size matches that of the previous MRSI investigation in this patient group (Endres et al., 2025), although the group composition slightly differs. The patient cohort was heterogeneous, encompassing diverse antibodies, clinical profiles, disease stages, and medication regimens that may have shaped the results. Overall, 79 % had novel CNS antibodies, and only 21 % had well-characterized brain antibodies, though 86 % of all patients tested positive for CSF antibodies—an outcome not expected in healthy controls but not explicitly examined in them (as the controls did not receive a lumbar puncture). There remains a risk that certain patients were false positives in terms of suspected AP, particularly in cases if routine CSF testing was negative and novel CNS antibodies were of uncertain significance. Furthermore, the subacute onset criterion (<3 months), as suggested in the Pollak criteria (Pollak et al., 2020), was no inclusion criterion. Nonetheless, CNS antibody-positive patient groups who undergo comprehensive multimodal diagnostic work-up, including EEG, MRI, CSF, and often FDG-PET examinations, remain extremely rare in the literature. Indeed, a systematic review identified only 145 published cases, including children, adolescents, and patients with incomplete diagnostics (Endres et al., 2022b). In summary, this is the first study on functional connectivity in suspected AP spectrum syndromes. The results could have been influenced by factors such as previous immunotherapies, body mass index, or sociodemographic characteristics. Further measures of even low-grade inflammation such as C-reactive protein and pro-inflammatory interleukins/chemokines from serum/CSF were not analyzed. Given these limitations, the findings should be considered preliminary. Future fMRI investigations should focus on more homogeneous psychiatric patient cohorts (e.g., those with the same antibody, symptoms, and disease stage, and ideally unmedicated). If characteristic functional connectivity signatures can be established, such measurements might become less invasive biomarkers for diagnostic purposes.

## 5 Conclusion

This MREG/fMRI study showed that at least insular functional connectivity is altered in patients with suspected autoimmune psychosis spectrum syndromes, underscoring that alterations in functional connectivities could exceed those pathologies detected via conventional MRI. Further studies in more homogeneous cohorts will be crucial to validate whether specific patterns can be linked to particular CNS antibodies, potentially guiding biomarker-driven diagnostic approaches. In addition, machine-learning approaches could be applied in the future to simultaneously analyze all measurable advanced MRI/EEG data, psychometric/neuropsychological test results as well as laboratory findings from serum and CSF in order to identify signatures of autoimmune causalities in different psychiatric syndromes.

## CRediT authorship contribution statement

**Katharina von Zedtwitz:** Writing – original draft, Visualization, Validation, Software, Project administration, Methodology, Investigation, Formal analysis, Data curation, Conceptualization. **Ludger Tebartz van Elst:** Writing – review & editing, Supervision, Resources, Methodology, Funding acquisition, Conceptualization. **Isabelle Matteit:** Writing – review & editing, Investigation, Data curation. **Andrea Schlump:** Writing – review & editing, Project administration, Methodology, Investigation, Formal analysis, Data curation. **Thomas Lange:** Writing – review & editing, Supervision, Software, Resources, Methodology, Conceptualization. **Kimon Runge:** Writing – review & editing, Data curation. **Judith Weiser:** Writing – review & editing, Formal analysis. **Kathrin Nickel:** Writing – review & editing, Methodology, Data curation. **Katharina Domschke:** Writing – review & editing, Supervision, Resources. **Harald Prüss:** Writing – review & editing, Supervision, Investigation, Data curation. **Alexander Rau:** Writing – review & editing, Methodology, Formal analysis. **Marco Reisert:** Writing – review & editing, Software, Methodology, Formal analysis. **Simon J. Maier:** Writing – review & editing, Supervision, Methodology, Investigation, Formal analysis, Data curation. **Bernd Feige:** Writing – review & editing, Visualization, Supervision, Software, Resources, Project administration, Methodology, Investigation, Funding acquisition, Formal analysis, Data curation, Conceptualization. **Dominique Endres:** Writing – review & editing, Supervision, Resources, Project administration, Methodology, Funding acquisition, Data curation, Conceptualization.

## Ethics/consent for publication

The study received approval by the Ethics Committee of the University Medical Centre Freiburg (no: 209/18). All participants gave signed written informed consent for participation in this study.

## Funding

The study was funded by the 10.13039/501100001659German Research Foundation (Project-Nr.: 419859038).

## Declaration of competing interest

KD: Member of the Neurotorium Editorial Board, The Lundbeck Foundation. She received speaker's honoraria by Janssen-Cilag GmbH. LTvE: Advisory boards, lectures, or travel grants within the last three years: Roche, Eli Lilly, Janssen-Cilag, Novartis, Shire, UCB, GSK, Servier, Janssen, and Cyberonics. All other authors declare no potential conflicts of interest.

## Data Availability

Data will be made available on request.
